# Datasets on factors influencing career planning among Chinese university students based on the theory of planned behavior

**DOI:** 10.1016/j.dib.2026.113181

**Published:** 2026-08-22

**Authors:** Qing Sun, Xiaohui Liu, Meng Wang, Heming Yang

**Affiliations:** a College of Forestry and Grassland Science, Jilin Agricultural University, Changchun 130118, China; b Jilin Province Agricultural Technology Extension Station, Changchun 130033, China; c College of Geographic Science and Tourism, Jilin Normal University, Siping 136000, China

**Keywords:** TPB, College students, Career planning, Influencing factors, Dataset

## Abstract

This dataset presents university students’ career planning goals and their influencing factors. A questionnaire was developed based on the Theory of Planned Behavior (TPB), and data were collected from 1,400 students across 28 provinces and 12 academic disciplines in China. The dataset includes measures of perceived behavioral control, attitudes, subjective norms, demographic characteristics, and career planning goals. Descriptive statistical analyses were conducted to summarize the data and examine correlations between career planning goals and subjective factors. Furthermore, this dataset provides valuable insights for university career education and supports future comparative research on career planning choices across different academic majors.

Specifications Table

**Table:** 

Subject	Social Sciences
Specific subject area	*College students' career planning goals and related views*
Type of data	Table (.xlsx format)
Data collection	*The target population for this survey comprised university students in China. Data were collected through an online questionnaire completed by 1400 university students. The data collection period spanned from November to December 2025*.
Data source location	*Region: Asia Country: China*
Data accessibility	Repository name: Dataset of Factors Influencing Career Planning of Chinese University Students Data identification number: 10.17632/fpgdsvm2gs.1 Direct URL to data: https://data.mendeley.com/datasets/fpgdsvm2gs/1
Related research article	*None*

## 1 Value of the Data

• This dataset provides substantial value to educational researchers seeking to understand the factors influencing university students’ career planning. It enables researchers to examine whether students with different career goals exhibit distinct psychological and social characteristics and whether demographic factors are associated with these patterns.
• The dataset includes multiple indicators of attitudes, subjective norms, and perceived behavioral control, enabling researchers to examine which specific dimensions are most strongly associated with students’ career planning goals. The dataset also facilitates the formulation of hypotheses concerning the psychological and social mechanisms underlying students’ career decision-making and provides a foundation for subsequent empirical and longitudinal studies.
• The dataset is particularly well suited for confirmatory analyses, including structural equation modeling, regression analysis, mediation and moderation analyses, and subgroup comparisons. Additionally, the dataset supports predictive and classification approaches, including machine learning techniques, to identify patterns associated with different career planning goals.
• The dataset includes students from 12 academic disciplines, enabling researchers to examine differences in career planning goals and TPB-related factors across disciplines within the study sample. This enables researchers to identify potential discipline-specific patterns and formulate hypotheses for future multi-institutional or nationally representative studies.
• Furthermore, the dataset can be used by university administrators and career education practitioners to identify students’ career planning needs and design targeted career education activities. It can also help identify student groups that may benefit from targeted career counseling or career information services.

## 2 Background

Career planning among university students is of paramount importance in today’s socioeconomic and educational context. With the expansion of higher education and rapid global economic development, university students face increasingly intense competition in the job market as well as growing uncertainty in their career development [1]. Effective career planning is not only a cornerstone of individual students’ career success but also a crucial foundation for optimizing the social talent structure and promoting sustainable development. It encourages university students to systematically reflect on and design their future career paths based on their interests, abilities, values, and societal needs, thereby enabling informed career decision-making and effective implementation [2].

University career education, as an integral component of the higher education system, plays a more substantial role than traditional employment guidance [3]. It helps students develop positive career values and enhance career adaptability and self-efficacy, thereby strengthening their resilience in the face of career changes. By offering personalized career counseling, mock interviews, and internship recommendations, career education effectively addresses students’ limitations in career information acquisition and decision-making, reduces uncertainty in career decisions, and enhances confidence in career choice [4]. By cultivating students’ professional competencies, entrepreneurial awareness, and lifelong learning capabilities, this approach directly supports the national talent strategy and the innovation-driven development strategy [5].

Existing research has examined a wide range of factors influencing college students’ career planning, including individual psychological traits, sociodemographic characteristics, and external environmental factors (e.g., career exploration behaviors, school support policies, teacher–student relationships, peer relationships, training programs, and institutional facilities) [1,2]. However, limitations remain in elucidating the intrinsic mechanisms underlying these influencing factors, particularly with respect to their systematic integration into established psychological theoretical frameworks. Few studies have incorporated these factors into classic behavioral theories, such as the Theory of Planned Behavior (TPB), for in-depth analysis. The Theory of Planned Behavior (TPB) [6,7], a widely used framework for explaining human behavior, posits that individuals’ behavioral intentions are jointly determined by attitudes toward the behavior, subjective norms, and perceived behavioral control [8]. This study aims to address this research gap by systematically collecting and organizing data on factors influencing the career planning of Chinese university students and integrating these factors with the TPB framework for in-depth analysis. Using the TPB theoretical framework, this study re-examines factors influencing career planning identified in previous research, with the aim of more comprehensively elucidating how these factors shape university students’ career attitudes, subjective norms, perceived behavioral control, and, ultimately, their career planning goals and behaviors.

## 3 Data Description

These data were collected through an online survey of 1400 students from public and private universities across 28 provinces in China. The survey employed a questionnaire that included demographic information. The dataset includes respondents’ career planning goals (civil service examinations, postgraduate entrance examinations, and employment), perceived behavioral control (e.g., stress tolerance, problem-solving skills, and planning ability), attitudes (e.g., income expectations, pursuit of social status, and confidence in goal attainment), subjective norms (e.g., social support, teacher advice, and peer influence), and demographic characteristics (e.g., gender, grade level, major, and student leadership status).

**Fig. 1:**
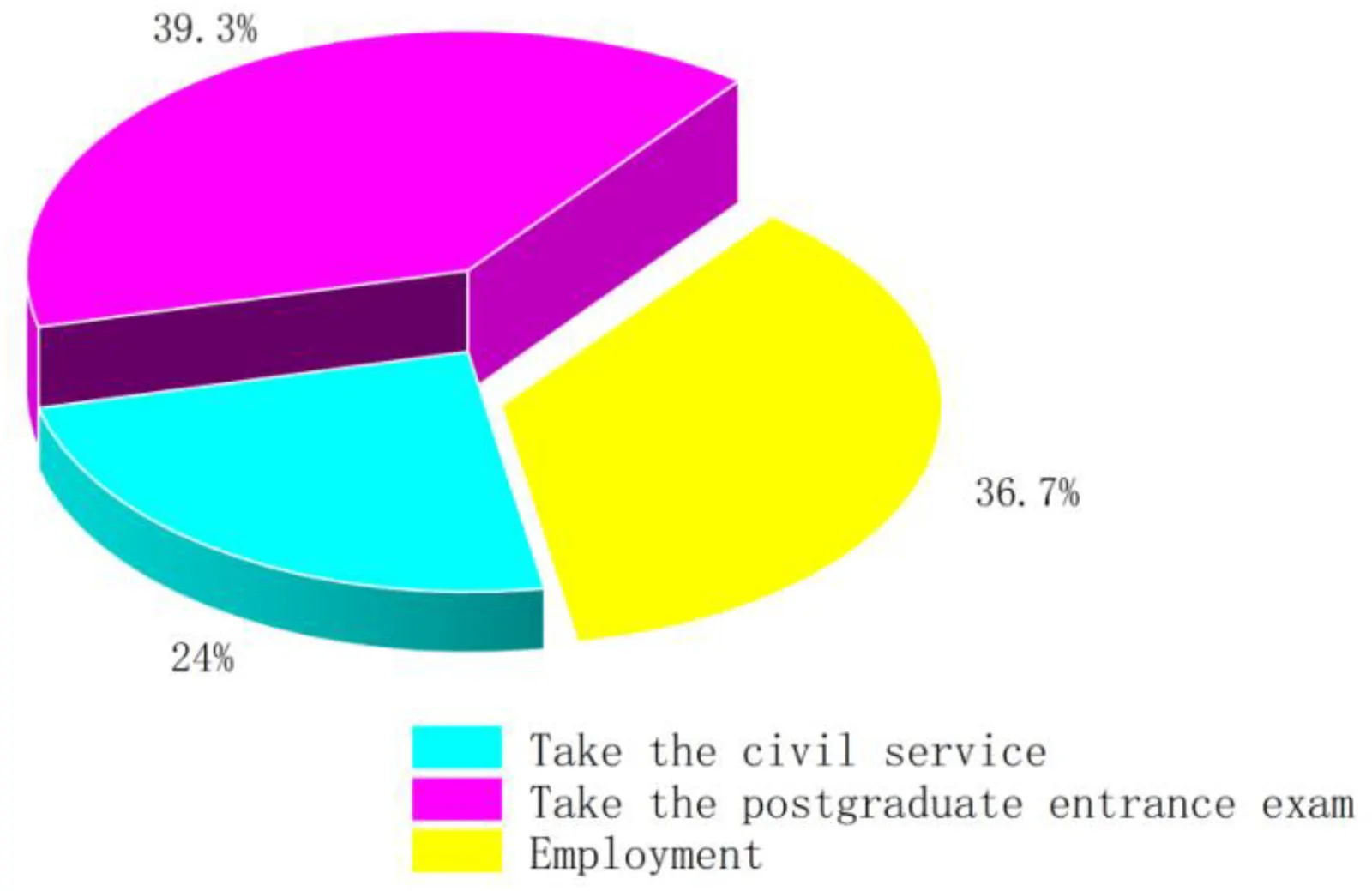
Distribution of career planning goals.

**Fig. 2:**
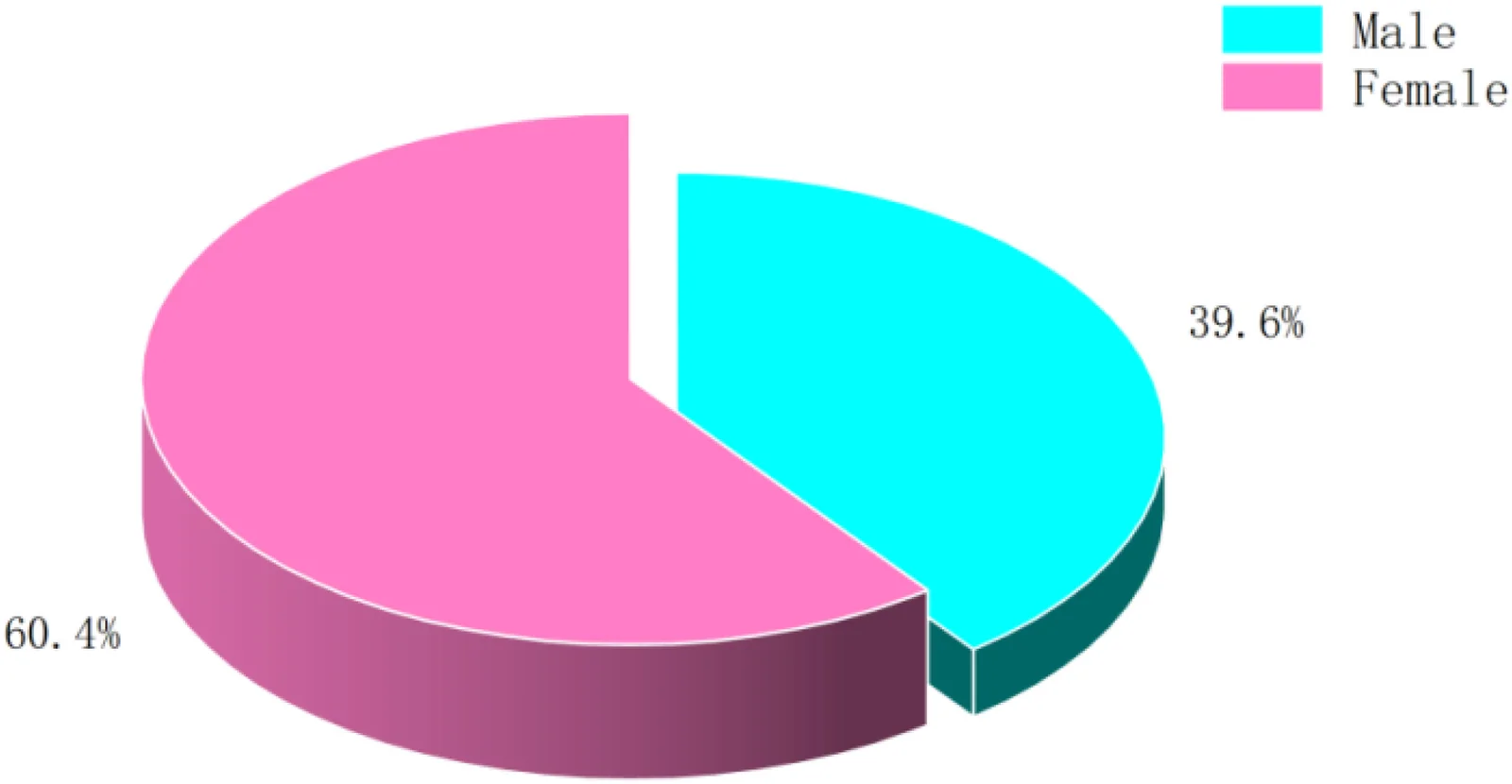
Gender distribution of respondents.

**Fig. 3:**
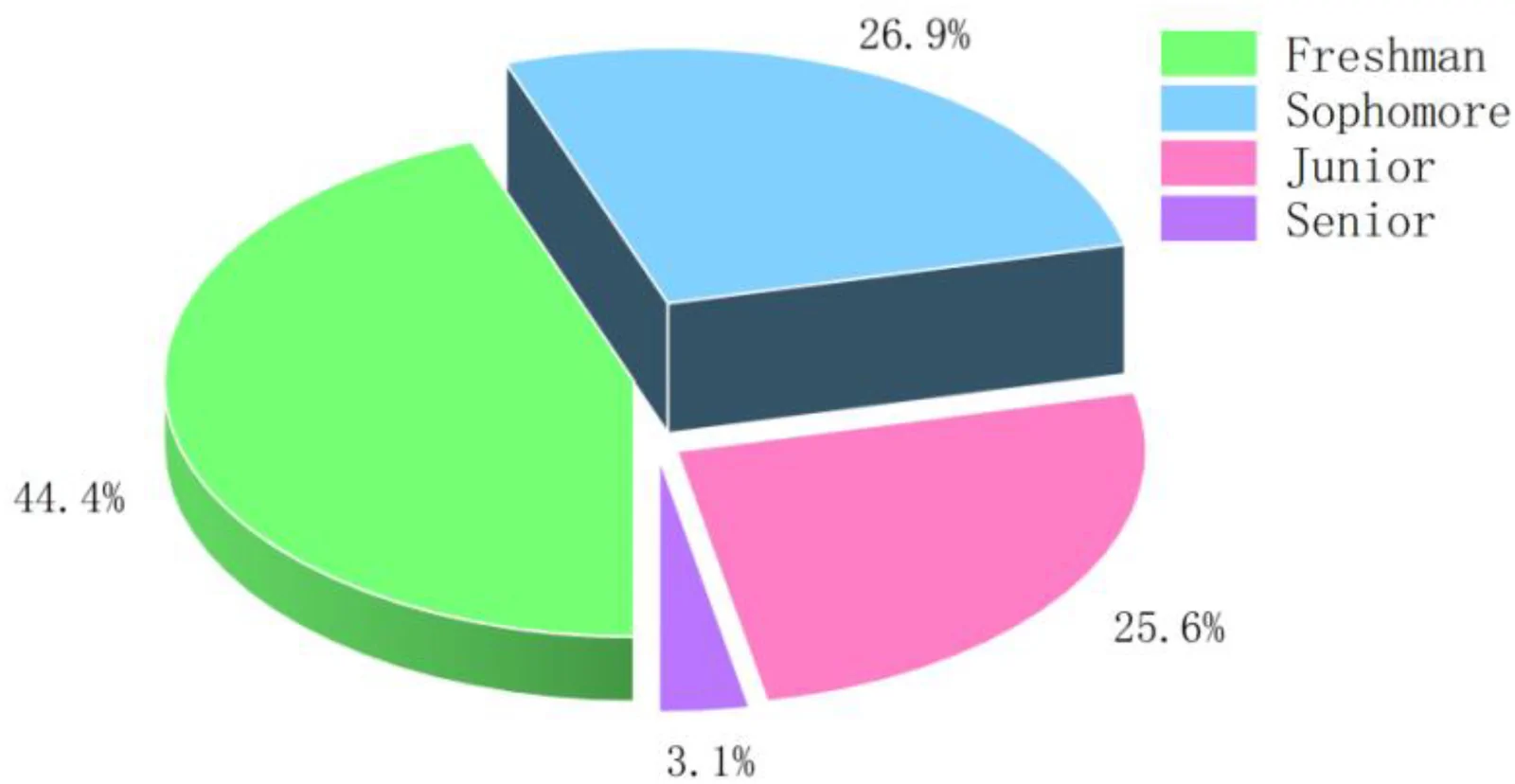
Distribution of respondents by university year.

**Fig. 4:**
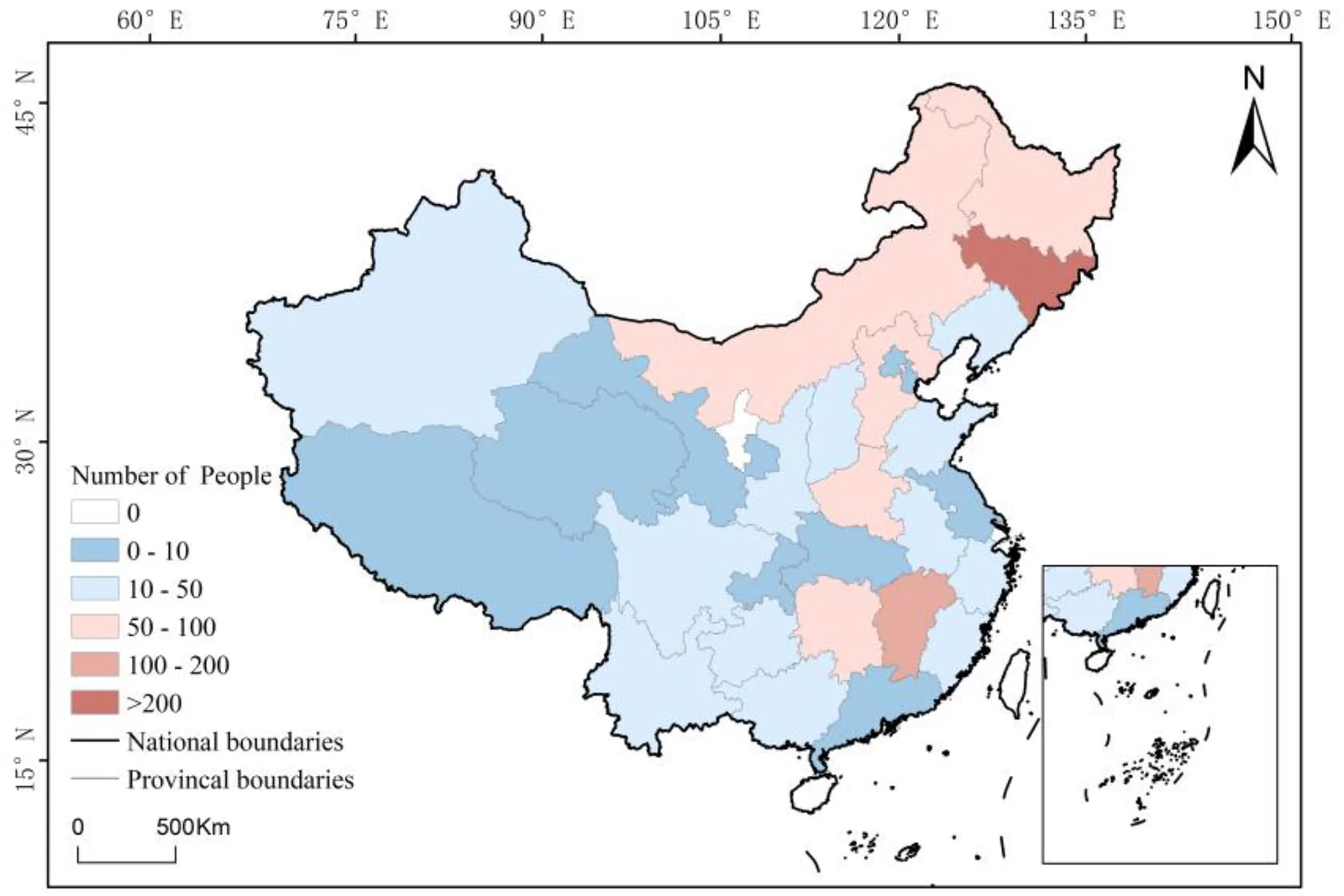
Distribution of respondents' hometowns.

Fig. 1 illustrates the distribution of career planning goals, showing that most respondents selected postgraduate studies (39.3%) and employment (36.7%). Fig. 2 presents the gender distribution, indicating that 60.4% of respondents were female. Fig. 3 presents the respondents’ university year, showing that first-year students comprised the largest group (44.4%). Fig. 4 illustrates the distribution of respondents by their place of origin, with most respondents originating from Jilin, Jiangxi, and Hebei provinces. These respondents represent 28 provinces, covering the majority of China.

## 4 Experimental Design, Materials and Methods

This questionnaire aims to investigate the factors influencing career planning among Chinese university students. Grounded in the Theory of Planned Behavior (TPB), the instrument incorporates demographic information and career-related background variables and draws upon established scales from previous research [4,9], with localized adaptations to better reflect the context of Chinese university students. All items are rated on a five-point Likert scale, ranging from 1 (``strongly disagree'') to 5 (``strongly agree''). Three TPB-related constructs are measured: attitudes, subjective norms, and perceived behavioral control.

Attitude. This construct refers to students’ evaluations of career planning outcomes and their perceptions of the importance of various characteristics of future employment and career development. The attitude items cover aspects such as expected income, job stability, social status, and work–life balance. A representative item is: “How important do you consider the expected income of your future job to be?”

Subjective Norms. Subjective norms reflect the social influences that may shape students’ career planning decisions. The measured dimensions include peer influence, the economic conditions of students’ hometowns, teacher advice, and perceived social pressure. A representative item is: “To what extent do you feel influenced by the social pressure associated with a career?”

Perceived Behavioral Control. Perceived behavioral control refers to students’ perceptions of their ability and available resources to manage and implement career planning decisions. The measured dimensions include stress tolerance, problem-solving skills, resource acquisition capabilities, time management skills, and career planning competence. A representative item is: “How would you rate your problem-solving ability?”

Participants were recruited through student social media platforms at multiple universities. The survey was conducted from November to December 2025. Before completing the questionnaire, participants were informed of the study’s purpose, estimated completion time, and the confidentiality of their responses. Participation was entirely voluntary, and no monetary compensation or other direct benefits were provided. A total of 1451 participants completed the questionnaire. After screening out incomplete or non-compliant responses, 1400 valid questionnaires were retained for analysis. Participants were assured that all income-related information would be kept strictly confidential and used solely for academic research purposes.

The collected data were stored in an Excel file, coded, and imported into IBM SPSS Statistics version 27 for analysis to assess data standardization based on the consistency of standardized response scores. Table 1 presents the results of descriptive statistical analyses, including the mean, standard deviation, frequency distribution, and reliability of the survey instrument, assessed using Cronbach’s alpha. The reliability assessment aimed to evaluate the internal consistency of the measured constructs and confirm their adequacy.

**Table 1:** Descriptive statistical analysis.

Category	Abbreviation	Statement	Mean	S.D	Factor loadings	Cronbach’s Alpha	Composite Reliability	KMO	Bartlett's test	Average Variance Extracted
Attitude	ATT_Income	Importance of Expected Income	4.34	0.804	0.643	0.774	0.775	0.759	0.000	0.463
	ATT_Stability	Importance of Job Stability	4.30	0.792	0.634					
	ATT_Status	Importance of Social Status	3.69	0.894	0.698					
	ATT_Balance	Importance of Work-Life Balance	4.09	0.887	0.741					
Perceived Behavioral Control	PBC_StressTolerance	Stress Tolerance	3.31	0.998	0.757	0.895	0.896	0.866	0.000	0.633
	PBC_ProblemSolving	Problem-Solving Ability	3.42	0.949	0.819					
	PBC_ResourceAccess	Resource Acquisition Ability	3.18	0.979	0.829					
	PBC_TimeManagement	Time Management Ability	3.21	0.987	0.782					
	PBC_CareerPlanning	Career Planning Ability	3.05	1.010	0.789					
Subjective Norms	SN_Peers	Peer Influence	2.88	1.003	0.598	0.742	0.744	0.721	0.001	0.422
	SN_Hometown	Influence of Hometown Economy	3.23	0.973	0.613					
	SN_Teacher	Influence of Teacher's Advice	3.16	0.907	0.661					
	SN_PublicOpinion	Influence of Social Pressure	3.02	0.952	0.719					

**Fig. 5:**
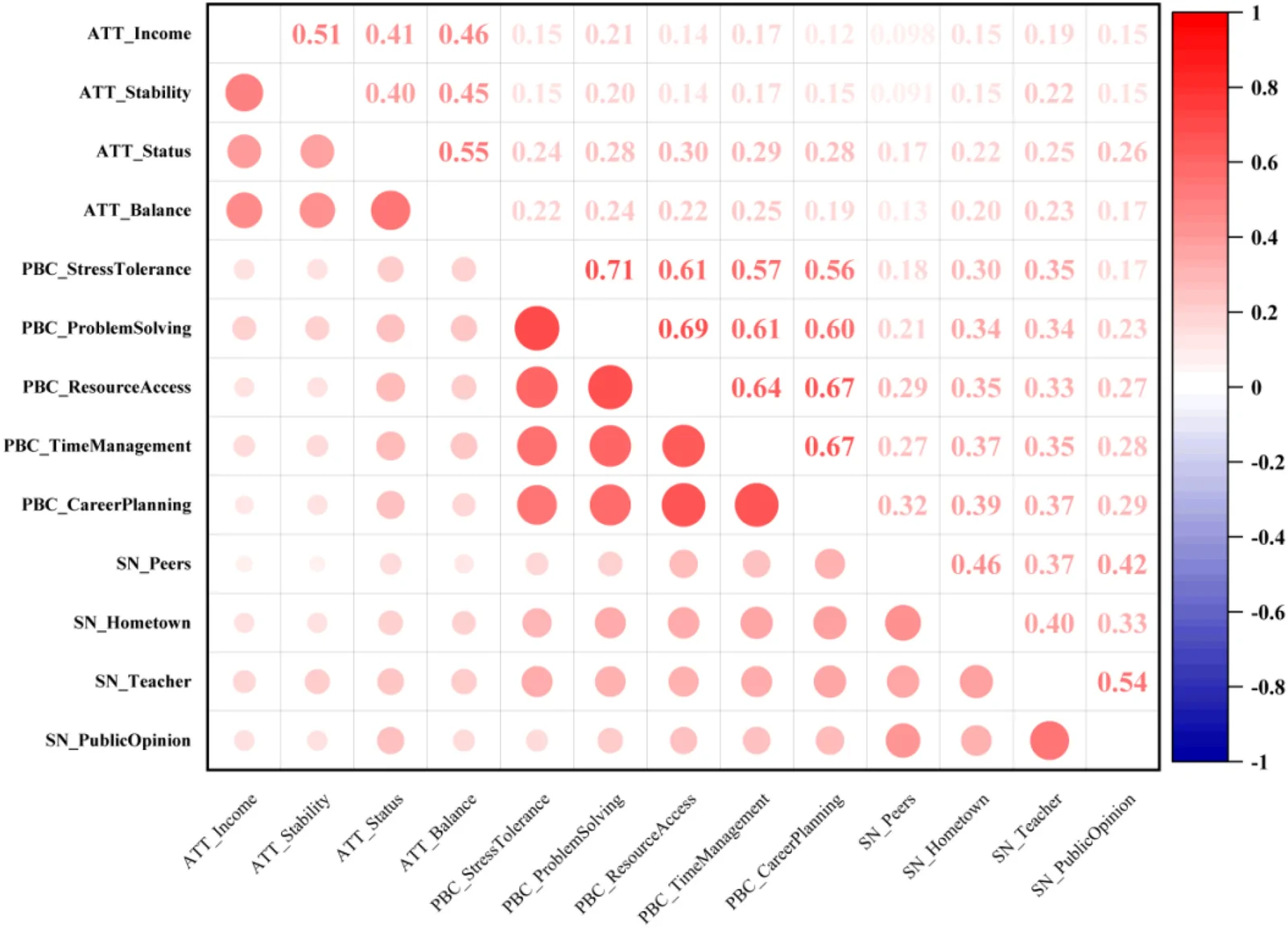
Correlation heatmap.

In addition, quantitative analyses were conducted using Origin to examine the relationships among respondents’ attitudes toward career planning, subjective norms, and perceived behavioral control. Fig. 5 presents a correlation heatmap, illustrating the strength and direction of associations among the variables.

## Limitations

This dataset has several limitations. First, regarding geographical coverage, the sample primarily includes students from 28 provinces and 12 majors in China, which may limit generalizability to other regions or countries. Second, the range of variables is limited. While the dataset focuses on factors such as demographic characteristics, perceived behavioral control, attitudes, and subjective norms, it overlooks important influences, including learning literacy, school support, and social assistance. It also reflects only students’ subjective views at the time, failing to capture potential trends or long-term changes. Finally, as the dataset relies entirely on quantitative survey data and lacks a qualitative perspective, it cannot provide in-depth background information to fully understand students’ career planning decisions.

## Ethics Statement

The authors have read and adhered to the ethical requirements for publication in Data in Brief. Prior to obtaining participants’ consent, all information regarding the research objectives, eligibility criteria, and expected response time was provided. Consequently, participants received full information and provided voluntary consent. Furthermore, to protect participant privacy, no personal information, such as names or identification details, was collected. It is hereby declared that the study adhered to the ethical principles of the Declaration of Helsinki and the ethical requirements for publication in *Data in Brief.*

## CRediT Author Statement

**Qing Sun:** Conceptualization, Methodology, Writing – original draft, Visualization; **Xiaohui Liu:** Conceptualization, Methodology, Writing – original draft, Visualization; **Meng Wang:** Data collection, Writing – original draft; **Heming Yang:** Data collection, Writing –original draft.

## References

[1] Hu M., Liu X., Yu X. (2026). Protean career attitude and career decidedness among Chinese college students: the mediating role of career planning and the joint moderating roles of career stress and impostor phenomenon. Int. J. Educ. Vocat. Guid..

[2] Zhang Z., Yu X., Liu X. (2022). Do I decide my career? Linking career stress, career exploration, and future work self to career planning or indecision. Front. Psychol..

[3] Zega F., Giatman M., Irfan D. (2025). Implementation of the theory of planned behavior (TPB) in student career planning at Nias regional private vocational high school. J. Penelit. Pendidik. IPA.

[4] Study on the management of Chinese college students based on the perspective of career planning J. Educ. Educ. Res., (n.d.). https://drpress.org/ojs/index.php/jeer/article/view/15678 (accessed February 10, 2026).

[5] Manongsong M.J. (2025). Career planning, guidance and innovation among Chinese college students. Asia Pac. J..

[6] Duan W., Jiang G. (2008). A review of the theory of planned behavior. Adv. Psychol. Sci..

[7] Dong S., Chang Y. (2024). The theory of planned behavior explored in entrepreneurial intentions among university students in Shandong province, China. J Infrastruct. Policy Dev..

[8] Yang D., Zhang X., Zhang X. (2022). Proceedings of the 4th International Conference on Modern Education and Information Management, ICMEIM 2023.

[9] Gu T., Gai X., Wang Y., Jia F. (2023). Data report on career adaptability, personal value, and motivational interview among at-risk Chinese college students. Data Br..

